# Opportunities and challenges of indigenous biotic weather forecasting among the Borena herders of southern Ethiopia

**DOI:** 10.1186/s40064-015-1416-6

**Published:** 2015-10-15

**Authors:** Desalegn Yayeh Ayal, Solomon Desta, Getachew Gebru, James Kinyangi, John Recha, Maren Radeny

**Affiliations:** Department of Geography and Environmental Studies, MARIL and Debre Berhan University, Debre Birhan, Ethiopia; Managing Risk for Improved Livelihood (MARIL), Addis Ababa, Ethiopia; Climate Change Agriculture and Food Security (CCAFS), Nairobi, Kenya; Addis Ababa University, P.O. Box 150129, Addis Ababa, Ethiopia

## Abstract

The practical utilization of available modern as well as traditional weather forecasting systems builds herders’ resiliency capacity to climatic shocks. The precision and reliability of the forecasting system determines its creditability and acceptance by the users to be proactive in the decisions they make based on the forecasted information. It has been postulated that traditional weather forecasting systems are becoming less reliable due to repeated faulty forecasts. The study assesses the current status of the Borana traditional weather forecasting system and how traditional experts make weather forecasts based on biotic indicators such as intestinal readings, changes in plant and animal body languages. Questionnaire survey, field observations, focus group discussions and interviews with relevant key informants were employed to obtain data. Collected field data was compared with National Metrological Service Agency instrumental data for consistency. Results reveal that herders made short term weather forecasts using intestinal readings, and observed changes in plant and animal body languages. The study shows the extent how public confidence in the accuracy of indigenous weather forecasting skills has been gradually eroded overtime due to faulty forecasts. The precision and credibility of the traditional weather forecast steadily declined and led to repeated faulty predictions. Poor documentation, oral based knowledge transfer system, influence of religion and modern education, aging and extinction of traditional experts were identified as the major causes undermining the vitality of traditional climate forecast. Traditional weather foresting knowledge and skill could have some utility and also serve as a starting point to scientifically study the relationship between various signs and implied climatic events. This article recommends before traditional Borana weather forecasting system completely disappears, a remedial action should be carried out to rescue this long established wisdom, knowledge and skill and maximize the benefits from what works well. The forecast needs of herders could be rendered by a combination of modern and traditional weather forecasting services. Further research is required to explore possible area of complementarity between the modern and traditional forecasting systems for improved efficiency and effectiveness in predictability, dissemination and advice.

## Background

The idea and practice of indigenous weather forecasting is inbuilt in many cultures and has been established after long years of observation (Ziervogel and Opere 2010). Different cultures make use of biotic indicators to predict future weather conditions. For example, plant, human and animal conditions are used in Zimbabwe for weather forecasting (Shoko 2012; Joshua et al. 2012). In Nigeria and Kenya, observation and interpretation of animals’ body conditions and behaviors are employed to derive information about future weather conditions (Shukurat et al. 2012; Speranza et al. 2010; Ziervogel and Opere 2010). Similar practices exist among cultural and ritual specialists in Burkina Faso and Swaziland who also infer future weather conditions based on the physical signs of plants (Roncoli et al. 2001). There is much commonality of practices among different cultures.

However, recent studies have grown increasingly pessimistic about the viability of traditional weather forecasting mechanisms (e.g. Makwara 2013; Joshua et al. 2012). Among other things, the declining popularity of traditional forecasting is explained by the extinction of some biotic species that were used for weather forecasting (Kipkorir et al. 2010; Roncoli et al. 2001). The expansion of modern education and monotheistic religions is another factor undermining the claimed rationality of indigenous knowledge (Joshua et al. 2012). In many places, the precarious survival of indigenous weather forecasting skills is further undermined by poverty, lack of clear knowledge transfer mechanisms and poor documentation (Makwara 2013; Shoko 2012; Nakashima et al. 2012; Speranza et al. 2010; Chang’a et al. 2010). It is therefore necessary to study the status of indigenous practices of weather forecasting among the Borena before they vanish beyond possible recovery.

To date, modern science has not come up with a conclusive stance for or against the claims of indigenous weather forecasting although some believe that modern science could gain valuable insights from indigenous knowledge (Mundy and Compton 1991). The idea of integrating the experience of modern science and indigenous knowledge for more rigorous weather forecasting is valued. This is because weather information is critical to agricultural decisions pertaining to cropping time, stocking size and rangeland management (Tekwa and Belel 2009). In other words, the resilience of herders to climatic shocks is in part determined by the practical utility of available weather forecasting systems (Ekitela 2010; Doherty et al. 2009; Field 2005; Oba 1997). Accordingly, this study was conceived with a view to identify the inherent limitations of traditional weather forecasting among the Borena and to pave the way for a better system of weather forecasting through a possible integration of tradition and modern science.

The last similar research in Borena was conducted over a decade ago (Lusenoa et al. 2002). However, the research approach in and outside Borena has been largely descriptive with almost a total avoidance of a critical examination of the internal validity of the knowledge claims of traditional weather forecasting systems. This study extends the discussion on traditional weather forecasting practices by evaluating how traditional experts interpret various biotic signs from scientific point of view. Moreover, this study situates the changing role of traditional weather forecasting with broader cultural, economic, social and institutional framework. In other words, this study is justified because it invites future discussion to reassess the causes for the declining quality of indigenous weather forecasting systems.

## Study area and research methods

### Study area

This study was conducted in Borena zone among Borena herders where the CCAFS’s (Climate Change Agriculture and Food Security) learning site is located. Borena herders are the main inhabitants in the zone. The region is dominated by a semi-arid weather with a bi-modal rainfall regime ranging on average from 400 mm in the south to 600 mm in the north annually. About 59 % of the precipitation occurs from March to May and 27 % from September to November (ORDPEDB 2000). Pastoralism is the main economic activity in the study area.

### Methods

#### Sample size and sampling

Dikale and Dembelaseden in Yabello woreda and Alona and Gada in Arero woreda are Pastoral Associations in CCAFS (Climate Change Agriculture and Food Security) learning site were purposefully selected for the study. These PAs constitute some of the key areas for Borena ritual (tradition) as well the livestock production practices. Part of the sites and routes for the Gada seasonal movement and practices, the major grazing lands such as the Dida Hara plain and watering points are located in Yabello and Arero Woredas. For the questionnaire survey 50 households were selected using a simple random sampling method from each PA (Pastoral Association), which makes the total sample size 200. In consultation with the PA development agents, 36 male and 12 female household heads were selected for focus group discussion sessions. A total of six intestine readers locally called *Uchus,* eight veterinarians, and four Ethnobotanists were also interviewed.

#### Data sources and collection methods

In this research selected PRA tools, questionnaire survey, interviews were used to collect data. PRA (as a tool overt observation, focus group discussion) were used to collect data. A total of four focus group discussion sessions having an average of 12 participants’ each were organized to collect data on the precision of traditional weather forecasting system, herders attitude to biotic weather forecasting system, socio-economic preparation following weather forecasting. Overt observation was employed to understand how entrails, plant and animal body languages are interpreted by indigenous experts. Data was also gathered through in-depth interviews from Ethnobotany, veterinarian, Borena indigenous intestinal readers (Uchu) and community key informants. Questionnaire survey was employed to assess individuals’ socioeconomic preparations in view of weather forecasts and their perceptions towards the vitality of traditional weather forecasting systems. Data was collected from two slaughtered goat intestinal diagnostic. This approach was used to understand how *Uchus* interpreted different signs to weather events. Metrological data was also collected at National Metrological Agency to compare the historical recorded data with indigenous weather forecasted. For this research, data was collected between January and May 2013.

#### Data analysis

For the qualitative data that was collected during individual interviews, observation and focus group discussions a thematic content analysis was applied. The household survey was analyzed using a simple descriptive statistics i.e. percentage. The drought assessment method developed by Agnew and Chappel (1999) was adopted to identify drought and normal years within the last 27 years.

## Results and discussions

### Participants background information

Table 1 below provides a sociological profile of herders for this research. Illiteracy is widespread and attendance of school beyond the first cycle of primary education is only 0.5 %. A predominant majority of them (80 %) adhere to the traditional religion called *Wake Feta* followed by Islam and Christianity. Traditional weather forecast systems are the source of weather information for 96 % of respondents while the rest also have access to modern meteorological weather information. About 28 % of the participants are members for different types of credit and association showing a low level of livelihood diversification practice.

**Table 1:** Participants’ education, religion and access to weather information and credit

Items	Participants (N = 200)		
	M	F	Total
Educ. level			
Illiterate	167	25	192
1–4	7	0	7
5–8	1	0	1
9–10	0	0	0
Religion			
Wake Feta	142	17	159
Christian	10	0	10
Muslim	23	8	31
Others	0	0	0
Source of weather Information			
Indigenous sources	168	25	193
DAs	52	0	52
Relatives/Friends	165	21	186
Radio	21	0	21
TV	0	0	0
Credit Association Member	25	8	33

### Indigenous methods of weather forecasting among the Borena herders

#### Mode of acquiring the skill for weather forecasting

There are few individuals among the Borena herders who are conversant with one or more indigenous ways of weather forecasting. How the skill is acquired varies depending on the type of indigenous weather forecasting system. In general, skills that require extensive training and practice are inherited from fathers. It takes 1 or 2 years of training to master the craft of weather forecasting through intestine reading and someone who has mastered that skill is known as Uchu. However, the fact that the training is given orally may hinder a robust knowledge transfer across generations (Garcia et al. 2009). On the other hand, the skill of weather forecast based on observation of the physical conditions of some plants and animals is commonly known by ordinary people and does not require training. Experts of intestine reading claim to be able to forecast the future weather condition and prospect of peace and conflict and the fortunes of humans and animals. The skill is preserved to men but women are not precluded from receiving weather forecast information. Although the gender divide is obvious and exists in other places too (Berkes 2009; Turner and Clifton 2009), it is not clear how the interest of women are affected.

#### Indigenous mechanisms of weather forecasting

##### Reading of animal behaviors and body conditions

It is indigenously believed among the Borena that specific body conditions and behaviors of animals during resource abundant time give clues about the future weather. A future drought is forecasted if cattle display the following behaviors. They become calm and sleep in the pen very close to one another. They refuse to go and graze in a nearby pasture while preferring to stay near water points after drinking. They lose appetite for grass and salt, not only do bulls lack interest in mating but also isolate themselves from the herd by staying in the nearby bush and showing reluctance to return to the pen. Likewise, other cattle body conditions regarded as signs of a future drought are: They become thinner with erected skin hair; their bellies never look full no matter how well they are fed; they defecate and urinate while in sitting position; and the amount of dung becomes smaller although they consume enough like other times.

To the contrary, the following cattle body conditions and behaviors are regarded as signs of a normal rainy season. They lick each other’s body, wander around villages in search of bone to eat, they display a relaxed mood and get away from water points after drinking. They show normal sexual desire and the bull visits many cows within a short period of time. The popular belief is that the above body conditions and behaviors, if exhibited when cattle are not sick or hungry, are reflections of future weather conditions. A study report in Kenya reveals that some of these body conditions are interpreted similarly but disagrees that cattle fight over food in view of a future of drought (Speranza et al. 2010).

Veterinary scientists hold views that partially contradict and partially agree with indigenous explanations of the body languages and behavior of cattle. On the one hand, they seem to support indigenous views, when for instance, they noted that animals could naturally perceive and respond to the incoming weather condition, which is regarded as a central feature of survival strategy. In fact, some veterinarians are of the opinion that animals can perceive future natural phenomenon more sharply than modern technology sources. Thus, from a veterinary point of view to sensing future developments and making physiological and behavioral changes is a central element of animals’ survival instinct. For instance, cattle can prepare themselves for future harsh conditions by reducing their appetite for food and mating. Other veterinarians attribute animal behavior and body conditions, traditionally regarded as indicators of future drought, to disease and environmental stress.

There are also wild animals and insects whose behavior is observed for weather forecast. If a ground squirrel (*Tuka*) is busy digging holes a normal rainy season is expected and vice versa. When an army of ants moves along a course of nearly a straight line, normal rainfall season is anticipated while drought will be forecasted if they are dispersed in search of food. The migration of bees during the season of resource abundance from north to south is regarded as a signal of a future of drought while normal rainfall will be forecasted if they migrate in the reverse direction. A similar tradition is reported from Kenya suggesting the wider expanse of the practice observing bee behavior for weather forecasting (Speranza et al. 2010). Passivity of termites from gathering and storing food leads to a weather forecast for drought but if they are busy building hills, gathering and storing food, a normal rainfall season is expected. It seems that the correlation of the above behaviors with their corresponding weather forecast is derived from a belief that those living things behave in ways that ensure their survival. Among the Borena herders, the varying tones of hyena screaming and bird song are employed to make forecasts about different things. But it is not possible to describe the musical or vocal scale of different voices of hyenas and birds here. However, result is in line with previous studies by (Lusenoa et al. 2002) in Borena.

##### Reading of plant body languages

As noted above, weather forecasting based on observation of plant conditions is common knowledge among ordinary Borena herders. At present, people make weather forecasts by observing the flowering pattern of two plants locally known as Tedecha (acacia flowering tree with narrow leaves and black fruit pods, Acacia tortilis) and Ret (aloe tree, a plant with fleshy-toothed leaves). People expect rainfall a month after the flowering of such trees. However, a prospective drought season is expected if such trees bloom small amount of flowers and shed more quickly than under normal circumstances. Unlike the Borena the tradition in Kenya considers the delay in the time of flowering as indictor of prospective drought (Speranza et al. 2010).

When plants locally called *Hamesa* (Commiphora africana), Agarsu (Commiphora erythraea), *Dakkara* (Boswellia neglecta), and *Sukela* (Delonix elata) sprout lavish green leaves on the months of *Obra*-*Teka* (August) and *Amagiid* (January) a normal rainy season is forecasted for the small and main rainy seasons respectively. Otherwise, a drought will be forecasted for the next season. A plant type called Bisduga (*Kirkia burgeri*) is preferred for a more accurate weather forecast but the observed physical conditions and the corresponding interpretations are similar with the other plant types. Diverging in this research result Shoko (2012) reported that plants used for seasonal forecasting.

From scientific point of view, Botanists have contradictory assessment about plants’ ability to sense future climatic conditions and make the necessary responses. Addis Ababa University Ethnobotanists and Biomedical professors observed that plants have natural ability to sense the future climatic condition and adjust themselves in view of incoming climatic extremes. For instance, before drought occurs plants maximize their survival potential by reducing their food requirement through restricting their growth and shedding their flower before pollination thereby limiting their fruit production. Such physiological adjustments are regarded as viable coping mechanisms essential to conserve energy to be used in times of drought (see similar scientific arguments from Boko et al. 2007; Speranza et al. 2010). To the contrary, other botanists from Oromia research center argue that the said plant physical conditions have nothing to do with environmental conditions that would be experienced after days and months. That means, plant body conditions are expressions of the actual environmental conditions they actually live in rather than manifestations of future weather realities.

From the forecasting assumptions one may raise doubts about the vitality of plant based weather forecasting to prepare herders for meaningful adaptation. What is significant for drought forecasting is not the fact that those trees flower a month before the normally anticipated onset of rainfall, rather what is crucial is the amount of flower and their premature shedding. This means that, if trees really ‘foretell’ future weather, herders are left with only short time, about 2 weeks, between the shedding of flowers and the onset of normal rainy season to understand that drought is coming. It is questionable if herders can make viable adjustments with that short duration of forecasting capacity, although herders would suffer harshly without that forecasting capacity. On the other hand, herders have no control to distinguish whether or not the limited flowering and the early fall of flowers is caused by other factors such as disease or moisture stress. In view of the disagreement among Botanists and considering the limited reliability ascribed by herders to the accuracy of weather forecasting based on a reading of plant body language, it is recommended to conduct further research to understand the relationship between changing in plant characteristics and future weather conditions.

##### Reading of animal intestines

**Fig. 1:**
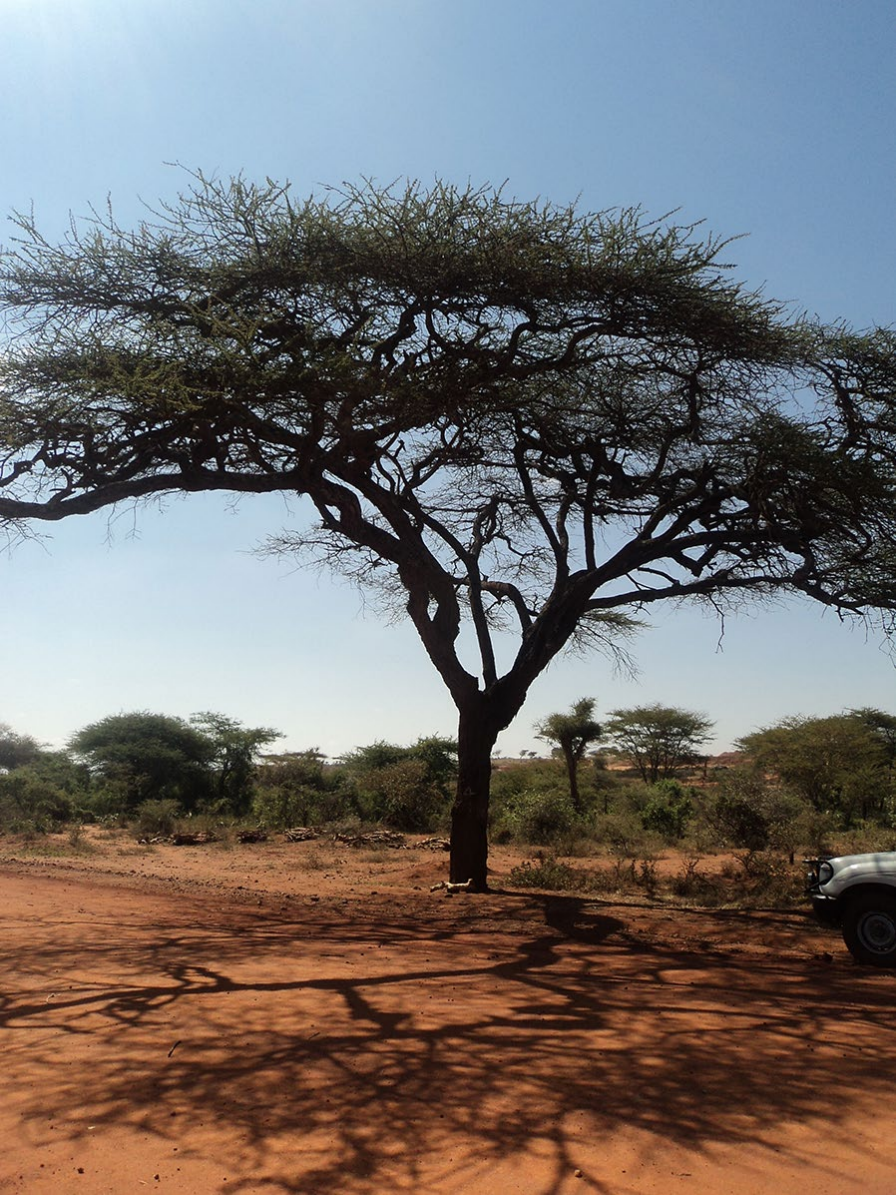
Tedecha (acacia) tree

**Fig. 2:**
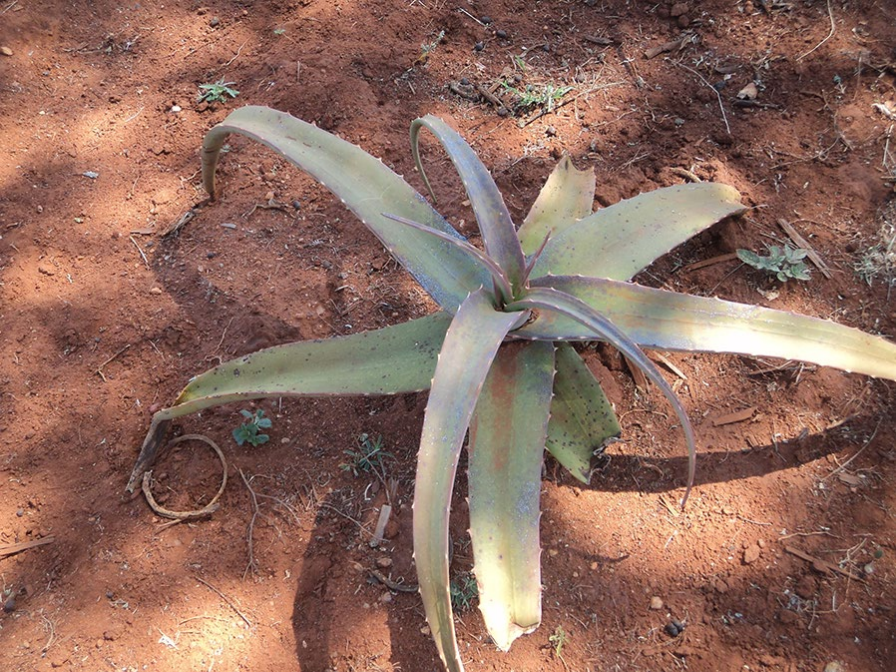
Ret (Aloe)

Unlike plant based weather forecasting, only a few indigenous ‘experts’, called *Uchu*, are considered to have the skill of reading different signs of animal intestines to forecast weather, social or individual fortunes and the prospect of peace and conflict from local to global levels. All domestic animals, except cats, dogs, chicken and equine are slaughtered for weather forecasting irrespective of their sex, type and age. However, a more precise forecast is believed to be possible from the intestine of a female animal, preferably cattle obtained from that locality. The reliability of weather forecasting based on intestine reading is second to indigenous astrology. Unlike the report of a previous study (Lusenoa et al. 2002) no unique ritual is practiced in slaughtering animals for weather forecast. Besides, there are no sexual or dietary restrictions on the Uchu to succeed in the reading and interpretation of animal intestines (Figs. 1, 2).

**Fig. 3:**
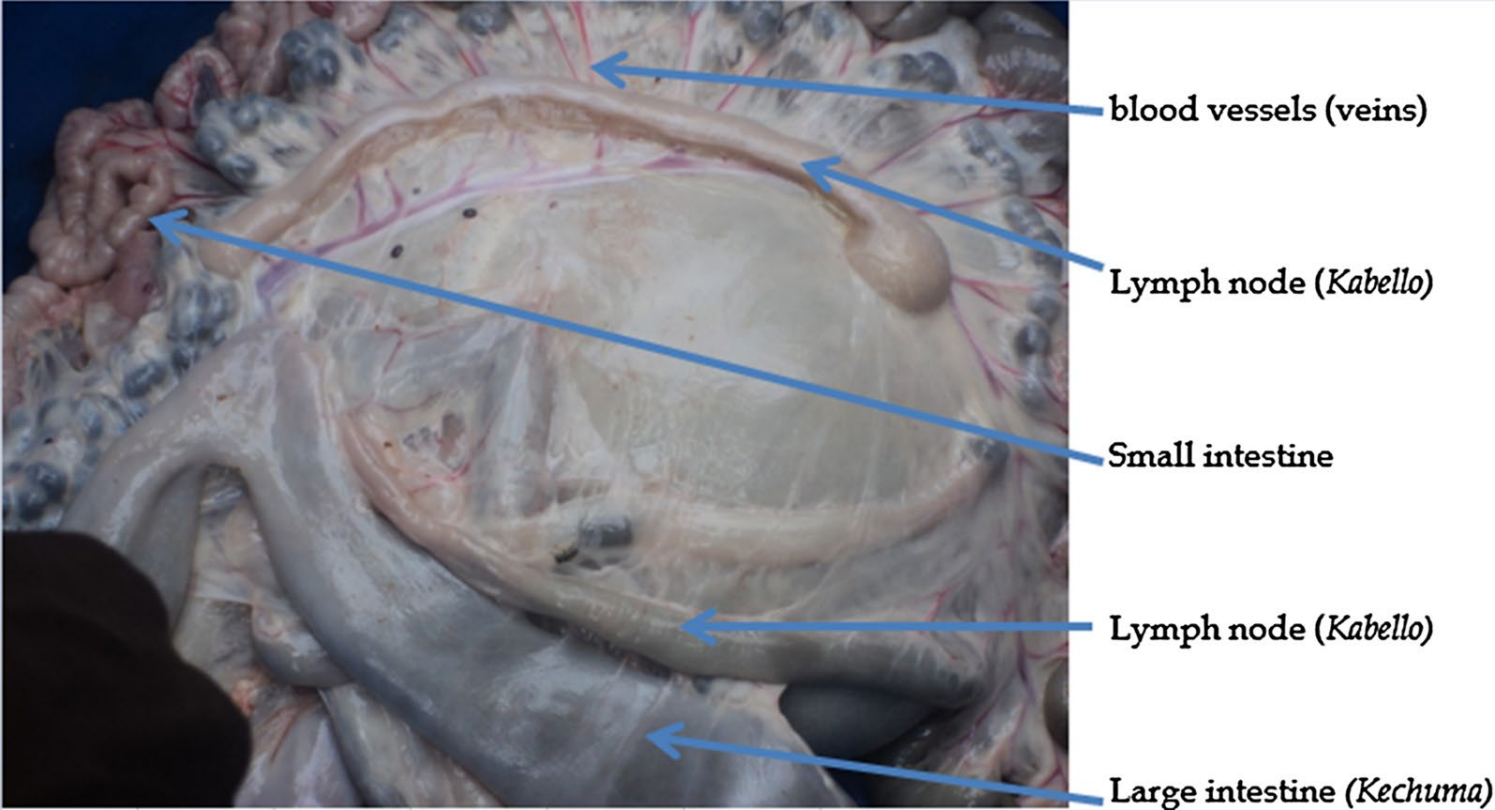
Parts of intestine used for weather forecasting

It is difficult to explain how intestine readings are used for weather forecasting since the skill is kept secret. However, from available information the practice of intestine reading and interpretation generally takes place like this: Indigenous experts look at four parts of the internal organ of animals. These are large intestine (*Kechuma*), small intestine, lump node (*Kabello*) and blood vessel (vein) (see Fig. 3). When the food substance, in the process of digestion, in the small and large intestines is small, medium and large, forecasts will be made for drought, small rainfall and a normal rainfall season respectively. Whether the forecasted drought would be mild or harsh is inferred in direct proportion to the size of food in the small and large intestines.

If the thickness of the lymph node is even throughout its parts and if the overlay tissue on the lymph node is darker in color, the next season is forecasted for a normal rain that would commence on time. Forecasting for drought is made if a very thin lymph node is observed. Regardless of its thickness, if a mark protrudes is observed at the end of the lymph node that is regarded as the right sign to forecast an outbreak of livestock disease. If experts observe a very thick lymph node and large intestine they forecast that rainfall will start after 2 weeks and 2 months respectively. The forecasted weather phenomena are believed to be experienced between 2 or 3 months from the date of intestine reading. One of the main advantage of intestinal weather forecasting over other modes of indigenous weather forecasting is that it helps to specifically indicate areas at the micro level according to the degree of rainfall or drought. Hence they make necessary arrangements.

The color and amount of blood observed on a certain section of the intestine (see Fig. 3) is another sign used for weather forecasting. When that vessel has good amount of darker color blood, experts anticipate bad rainfall in the coming season. On the contrary, if the vessel contains less blood with a yellowish color, the forecast for the next season would be normal rainy season. The severity of the drought is inferred in direct proportion to the amount of blood contained in the vessel; if no blood is seen the worst drought is expected.

During the field research, the researcher obtained similar interpretations on the intestine signs of two goats from two ‘experts’. In both cases, a normal rainy season was forecasted for the main rainy season of 2013 and it turned out to be true. All indigenous rainfall forecasting made for the previous main rainy season foresaw a normal rainy season and that turned out correct. Whether that is a mere coincidence or a proof of the accuracy of intestine based weather forecasting is difficult to comment based on that single observation. The forecasting for the rainy season that will commence in September: normal rain is forecasted using intestine reading while astrology based forecasts envision a delay in the onset of the rainy season and lower volume of rainfall.

What is the implication of those intestinal features in science? A few consulted veterinarians have an entirely different take on the causes of the above intestinal features and none of the signs are attributable to the influence of future weather conditions. The difference in the amount of food substance in small intestine and Kechuma could be because of the laying position of the animal when slaughtered. The variation in blood color could be the effect of disease or toxic grass that is mostly encountered in times of environmental stress. Proper red color simply reflects the good health status of the animal rather than a code for the future of rainfall.

## System of disseminating weather information and decisions

Indigenous weather forecast is the main source of meteorological information for the Borana herders since time immemorial. Experts of different modes of indigenous weather forecasting have non-obligatory responsibility to inform the people about their forecasting. None of them are paid monetarily for their service, although they consume the meat of the slaughtered animal with people around. The indigenous weather forecasting experts forecast and share information using well-organized Borena cultural networks. These experts communicate weather forecasting information to heads of *Raba Gada* members and elders who then disseminate weather information by calling the people for urgent meetings or by going to market places, water points and village neighborhoods. It is also possible for the interested person to get weather information directly from forecasters. The Gada leaders’ advice the community to prepare themselves to the forecast information such as to destock, livestock movement and use resources effectively and strengthen *bussa gonnoffa* (traditional support and resources sharing). Besides, the development agents and an NGO called AFD (Action for Development) are reported to have important contribution in getting the modern and indigenous weather forecast disseminated to the people.

Thought herders are severely affected by faulty forecasting, they still actively take necessary coping and adaptation measures in line with the forecasting. If drought is forecasted, they strengthen area enclosure through community bylaw, saving water and grass, preparing livestock medicine, storing hay, make arrangements for migration to less drought affected areas, destocking animals, reducing expenditure and changing schedules of social and cultural festivities such as wedding. If the forecast shows good weather, herders prepare land for crop cultivation, open the enclosure for grazing early and prepare for social and cultural ceremonies.

## Public attitude about the vitality of indigenous weather forecasting

As observed in Zimbabwe the expansion of Christianity religion had a negative impact on the acceptance of indigenous weather forecast practices in the study area (see Joshua et al. 2012). As Table 2 reveals, only followers of the indigenous *Wake Feta* religion have entirely no contempt for the indigenous weather forecasting system. They believe that the knowledge of indigenous weather forecasting is the gift of “God” to them. For that reason, they are highly cooperative in passing indigenous weather forecast information to others since they believe that the viability of their livelihood is intimately tied with the contribution of indigenous weather forecasting. Next to adherents of *Wake Feta*, Muslims are tolerant to indigenous weather forecasting; possibly, because Islam is very old to the area it is not opposed to tradition. Therefore, Muslims in Borena are not hostile to indigenous weather forecasting practices. It is among the Christians that indigenous weather forecasting is most rejected as the worship of idols. For Christians, indigenous experts (or they would prefer to call them witchdoctors) are guided by evil spirit that can neither tell nor change the future which is determined and known only by God.

**Table 2:** Community trust in traditional weather forecasting across religion

Religion	Wrong practice against God		Right practice	
	No. replies	%	No. replies	%
Wake feta	2	1.25	157	89.75
Christian	10	100	0	0
Muslim	7	22.5	24	77.4

Different indigenous weather forecasting systems have varying level of acceptance depending on their reliability (Speranza et al. 2010). This also applies to the Borena experience where the perceived reliability and hence acceptance of biotic weather forecasting methods in descending order is intestine reading, animal body language and plant body language. Today, however, informants stressed that people are increasingly losing confidence in all methods of indigenous weather forecasting which has led to the decline in the number of *Uchu*. The declining popularity of indigenous weather forecasting has sped up over the past 15 years. There has been a strong public reaction to the negative consequences people suffered due to the faulty indigenous weather forecasting. Most importantly, people are frequently affected when forecasting shows a normal rainy season but drought comes without preparation. *Uchus* repeatedly failed to tell the exact timing and intensity of rainfall and drought (Tables 3, 4).

**Table 3:** Herders’ response on the most reliable traditional weather forecasting indicator

Indicators	Most reliable indicator	
	No. replies	%
Intestinal reading	60	30
Animal body language reading	46	23
Plant body language reading	20	10

**Table 4:** Comparison of forecasting and percentage of mismatches with the reality

Year	Intestinal		Plant body language		Animal body language		Standardized precipitation anomalies		Counts of mach and mismatch in between different forecast	
	No. of replied	%	No. of replied	%	No. of replied	%	SPA value	Drought level	Mach/mismatch	Mach/mismatch
1985	51	26	23	12	19	9.5	0.42	Normal	*	***
1986	134	67	169	85	23	12	1.59	Normal	**	****
1987	74	37	58	29	93	47	1.01	Normal	*	***
1988	42	21	25	13	86	43	1.14	normal	**	****
1989	51	26	36	18	103	52	1.22	Normal	**	****
1990	51	26	39	20	27	14	0.35	Normal	*	***
1991	124	62	150	75	164	82	−1.2	Moderate	**	***
1992	123	62	168	84	170	85	0.23	Normal	**	****
1993	65	33	37	19	32	16	0.08	Normal	*	***
1994	119	60	33	17	186	93	−1.7	Extreme	**	****
1995	72	36	22	11	18	9	−0.8	Mild	**	***
1996	109	55	45	23	166	83	−0.3	Mild	**	****
1997	81	41	41	21	29	15	1.37	Normal	*	***
1998	63	32	33	17	102	51	−0.6	Normal	**	****
1999	52	26	26	13	24	12	−1.6	Moderate	**	***
2000	38	19	22	11	77	39	−1	Moderate	**	****
2001	158	79	173	87	83	42	−0.5	Mild	**	****
2002	53	27	15	7.5	100	50	−0.7	Mild	**	****
2003	8	4	11	5.5	11	5.5	−0.2	Mild	**	***
2004	121	61	50	25	150	75	0	Normal	**	****
2005	32	16	178	89	137	69	0.44	Normal	*	****
2006	103	52	24	12	176	88	1.2	Normal	**	****
2007	4	2	91	46	122	61	0.1	Normal	**	****
2008	114	57	0	0	0	0	−0.8	Mild	**	****
2009	13	6.5	0	0	0	0	−1.2	Moderate	**	***
2010	164	82	102	51	148	74	1.94	Normal	**	***
2011	123	62	164	82	112	56	0.47	Normal	**	****
2012	29	15	113	57	29	15	−0.9	Moderate	**	****
Counts of mismatch									22	17
Percentage of mismatch									78.5	60.7

* Mach of forecast between traditional methods and instrumental records

** Mismatch of forecast between traditional methods and instrumental records

*** Match of forecast within traditional methods

**** Mismatch of forecast within traditional methods

The assessment on the accuracy of indigenous weather forecasting made over the last 27 years justifies people’s tendency to downplay the accuracy of the system. More often than not, not only did different experts make different forecasts but many were found incongruent with the instrumental rainfall record as computed using Standardized Precipitation Index (SPI) where results below zero were taken to represent a condition of drought. For instance, a conflicting weather forecasting was made by different methods in 79 % of the studied years. The percentage of mismatch between indigenous weather forecasting and the actual rainfall record is 61 %. The increasing occurrence of forecasting errors over the last 27 years leads to the impression that indigenous weather forecasting is becoming an expression of a probabilistic weather regime. However, indigenous forecasters always insist that their forecasting is certain, a view strongly challenged by the public in recent years. In other words, the people are logical to question and sometimes ridicule the claims of indigenous weather forecast system.

Nonetheless, it would be premature to dismiss the intrinsic merits of indigenous weather forecast practices. Even in science, the stimulus-reaction pattern of things is generalized not from an absolute consistency of observed occurrences. That explains why scientific assumptions, theories and instruments cannot be fully relied on to explain every real life occurrences. The same allowance should be applied to the merits and demerits of indigenous weather forecasting mechanisms. People build traditions through a repeated observation of the stimulus-reaction patterns between and among variables. Because biotic clues were observed to correspond to a certain climatic regime since time immemorial, it would be outrageous to dismiss the reasoning of indigenous weather forecast systems because of their increasing dysfunction in recent years. Instead the integration of it with modern forecasting systems is essential and could advance the horizon of modern weather forecasting (Joshua et al. 2012).

For instance, cats, snakes, fish and monkeys respond to seismic waves before the occurrence of earthquakes but not always as if earthquakes can be forecasted from the behavior of those living things. Likewise, it could be possible that plants and animals could feel and respond to a future weather phenomenon but the frequency with which a similar stimulus-reaction is observed may not be reliable. At this stage, science has not reached the stage to confirm or to negate the existence of a connection between the body language and behaviors of plants and animals on the one hand and the future weather condition on the other. Accordingly, the wisdom of indigenous weather forecasting must await a conclusive scientific answer to the above challenges.

What is certain is that the accuracy of indigenous weather forecasting is dwindling from time to time for known and unknown reasons. Of the known factors, informants observed that some of the recent natural and social developments are jeopardizing the degree of accuracy with which different modes of indigenous weather forecasting operate. Acacia trees that have been largely used for a reliable weather forecast are disappearing. At the same time, different plant and animal species undergo behavioral changes in the face of ecological alterations. As a result, indigenous weather forecasts are confronted with a varying stimulus-reaction pattern. Of the social factors, the rising trend of alcoholism among the Borena in recent years is reported for the declining efficiency of indigenous experts. Any degree of intoxication is reported to have the impact of reducing the stamina of indigenous experts in enduring the painstaking task of meticulous observation and sober interpretation of different signs of the future weather.

## Conclusions

This study has shown that indigenous knowledge based weather forecasting is the main source of weather information in Borena where access to modern weather information is limited. Next to astrology, biotic means of indigenous weather forecasting is highly valued as a credible source of weather forecast. However, as in many other countries, the popularity of indigenous weather forecasting is declining for three main reasons. Firstly, the people are exhausted with the faulty weather forecasts and unbearable repercussions associated with it. Secondly, Christianity is growing in the area thereby discouraging the people from their allegiance to indigenous experts whom they equate with witchdoctors. Thirdly, influenced by modern education people are not attracted to indigenous knowledge which makes them ambivalent to study and uphold the indigenous science of weather forecasting. Other factors such as the passing away of indigenous experts, disappearance of indicative tress such as *Tedecha* (Acacia tortilis) trees, absence of documentation and oral transmission also play remarkable role. As a result, the number of indigenous weather forecast experts is declining at an alarming rate before adequate documentation is made, as observed in by other researchers (e.g. Joshua et al. 2012; Chang’a et al. Chang’a et al. 2010; Egeru 2012).

It is to be expected that the acceptance of indigenous weather forecasting could dwindle in the future. First, due to accelerated weather change and variability, plants and animals are changing their usual behavior. Furthermore, some of the biotic species used for weather forecast are disappearing and changing behavior because of climatic weather variability and disturbance of the ecosystem. As Egeru (2012) observed, in recent years the performance of indigenous weather forecasting is undermined by the changing nature of the behavior of biotic indicators. Against this background, indigenous weather experts may find it hard to make correct forecasts based on unusual body languages and behaviors of plants and animals. Moreover, the views of botany and veterinary do not endorse the reading and interpretations of biotic signs by indigenous experts. If indigenous weather forecasting, does not make strong appeal to scientific thinking, because science is not advanced enough to determine the truth or falsehood of indigenous assumptions of weather forecasting. However, influenced by the expansion of modern education and weary of the faulty forecasting, it is highly likely that people would increasingly relegate the tenets of indigenous knowledge of weather forecasting to superstition.

The challenge for indigenous experts is harmonizing weather interpretations with the changed stimulus-reaction patterns between different variables. In short, because of the repeated errors of indigenous weather forecasts and the erosion of indigenous values by modern education and Christianity, herders are likely to increasingly abandon their respect to indigenous weather forecasting and it is already happening. Therefore, the future of building herders’ resilience to climatic shocks depends on a better way of managing and integrating modern and indigenous weather forecasting.

Unlike a previous study (Lusenoa and his associates 2002), the Borena herders are not passive consumers of weather forecast information. Although the people are repeatedly misinformed by indigenous weather forecasters, they do make efforts to mitigate the effects of an expected drought through concert socio-economic responses that range from destocking to migration. Similarly, the claim that herders make ‘probabilistic forecasts, underscoring the fact that they acknowledge and accept forecasts of less-than-perfect skill’ (Lusenoa et al. 2002) is deviate in this research. Indigenous experts in Borena speak of their weather forecasts with high confidence rather in terms of probability. That explains why receivers of weather information respond in different ways.

In conclusion, from practical point of view the government and other relevant stakeholders should prepare exist strategy for indigenous weather forecast practices. They can no longer be sustained with the current level of inaccuracy without harming herders. A fertile ground already exists for the exit strategy as the people are largely disenchanted with the claims of weather ‘experts’. As tradition leaves its place for modern weather forecasting, effort should be made to lay the basic foundations that make accession of modern weather information by the herders possible. In the meantime, a better weather information management could be introduced. That is to say herders should be given both modern and indigenous forecast but advised to constantly live for the unexpected bad scenario.

From theoretical point of view, the scientific community, the government and other stakeholders should apply their joint effort to reassess the main causes for the declining quality of indigenous weather forecasting practices. At this stage, modern science has relative upper hand in providing weather forecasts over indigenous ways. However, the main limitation of modern weather forecasting is the very limited temporal scope for which generating relatively reliable weather information is possible. Notwithstanding the controversy over the vitality of indigenous weather forecasting, the system is far better than modern science when it comes to the longer duration covered in weather forecasting. If the true causes for the function and dysfunction of indigenous weather forecasting can be established, that would be a leap forward to the refinement and development of modern weather forecast technology. It is high time to forge collaboration between tradition and science for a better result.
